# Methodological Variations to Explore Conflicting Results in the Existing Literature of Masking Smile Judgment

**DOI:** 10.3390/bs14100944

**Published:** 2024-10-14

**Authors:** Annalie Pelot, Adèle Gallant, Marie-Pier Mazerolle, Annie Roy-Charland

**Affiliations:** 1 School of Psychology, Laurentian University, Sudbury, ON P3E 2C6, Canada; apelot@northernpsychologygroup.com; 2 École de Psychologie, Université de Moncton, Moncton, NB E1A 3E9, Canada; eag0327@umoncton.ca (A.G.); annie.roy-charland@umoncton.ca (A.R.-C.)

**Keywords:** enjoyment smiles, masking smiles, instruction, continuous rating scales

## Abstract

Although a smile can serve as an expression of genuine happiness, it can also be generated to conceal negative emotions. The traces of negative emotion present in these types of smiles can produce micro-expressions, subtle movements of the facial muscles manifested in the upper or lower half of the face. Studies examining the judgment of smiles masking negative emotions have mostly employed dichotomous rating measures, while also assuming that dichotomous categorization of a smile as happy or not is synonymous with judgments of the smile’s authenticity. The aim of the two studies was to explore the judgment of enjoyment and masking smiles using unipolar and bipolar continuous rating measures and examine differences in the judgment when instruction varied between judgments of happiness and authenticity. In Experiment 1, participants rated smiles on 7-point scales on perceived happiness and authenticity. In Experiment 2, participants rated the smiles on bipolar 7-point scales between happiness and a negative emotion label. In both studies, similar patterns were observed: faces with traces of fear were rated significantly less happy/authentic and those with traces of anger in the brows were rated significantly happier/more authentic. Regarding varied instruction type, no effect was found for the two instruction types, indicating that participants perceive and judge enjoyment and masking smiles similarly according to these two instructions. Additionally, the use of bipolar scales with dimensions between a negative emotion label and happiness were not consistently effective in influencing the judgement of the masking smile.

## 1. Introduction

Smile expressions are often considered universal signals representative of positive emotions such as happiness, the latter being one of the most accurately recognized emotion across different ages [1,2]. While a smile can be one of enjoyment when feeling happiness, the smile can also be utilized for other circumstances; they can be stimulated, amplified, minimized, neutralized, or to mask traces of emotions other than the one of happiness [3]. Thus, the judgment of an emotion for a decoder can be complexified. While trying to identify the emotion being expressed, noticing the subtilities that might reveal its sincerity or the true emotion vehiculated is crucial for effective communication.

However, the smile serves as a powerful masking strategy that individuals can use during experiences of negative emotions to conceal them (i.e., masking smiles) [4,5]. The masking strategies are not always flawless, and traces of the negative emotion escape into the smile expression producing micro-expressions [6]. Micro-expressions are subtle movements of the facial muscles or full flashes of a dissimulated emotion, manifested only in the upper or lower half of the face [5,7,8]. While micro-expressions can be very brief and be exhibited in low-intensity situations, they can also emerge when experiencing a strong emotional reaction [7,8]. Specifically, micro-expressions are generated, for instance, due to the difficulty associated with attempting to inhibit and control the facial muscles activated during the true experience of a negative emotion while simultaneously activating the facial muscles associated with the smile (Inhibition Hypothesis, [5,6,9,10,11,12,13,14]).

Despite these subtle movements, some research shows that individuals are sensitive to the enjoyment and masking of smile expressions [15,16,17,18,19,20,21]. In a study by Gosselin et al. [22], individuals’ abilities to distinguish between smiles that are characteristic of happiness and masking smiles that contained traces of anger were examined. They found that individuals as young as six years old could categorize the enjoyment smile expressions from the masking smile expressions. However, only the adults could accurately label the hidden emotion. Similarly, Perron et al. [20] and Pelot et al. [19] examined individuals’ sensitivity to enjoyment smiles and masking smiles that contained traces of anger, fear, sadness, and disgust presented in either the mouth or the eye area. Similar results were obtained: individuals were sensitive to the traces of negative emotions as accuracy exceeded chance levels in the judgment of these expressions as “not really happy” and in saying that there was another emotion present within the expression. Interestingly, differences were observed as a function of the negative emotions and where the trace of negative emotion was presented. For instance, participants were more sensitive for smiles with traces of fear, and least for anger when it was presented in the eye area than the mouth area. However, while these results show that participants are sensitive to the distinctive cues of enjoyment and masking smiles, other studies show that participants exhibit difficulty when asked to judge the sincerity of others’ expressions, with success rates of approximately 60% when the level of chance is at 50% [23,24,25]. Altogether, the studies’ different results clearly demonstrate inconsistencies regarding the judgment of enjoyment and masking smiles across the literature.

When reviewing the methodologies employed in the majority of the studies mentioned above as well as others, some striking limitations can be retained: (1) response measures were mostly dichotomous and (2) different wording was employed when instructing the participants to make a judgment on a smile. For instance, tasks that examined individuals’ judgments regarding masking smiles mostly measured the behaviour while only offering two answer choices (i.e., “really happy” vs. “not really happy”, yes/no question) [19,20,21,22,23,26,27]. In addition to employing dichotomous response measures, many tasks of smile judgment have assumed that having participants make judgments that categorize a smile as “really happy” or “not really happy” (e.g., [21,22]) is synonymous with having participants make judgments that categorize a smile as “authentic” or “not authentic”. (e.g., [28,29]). Therefore, it is worth questioning whether differences in these studies’ findings are due to differences in behaviour when judging smiles or to the methodological differences.

In effect, many studies have suggested that both dichotomous options and instruction type could have important implications on the results obtained. For instance, while a dichotomous rating method allows for an understanding about whether the expression is perceived as genuine happiness or not, it does not allow for an understanding of how the negative emotion is perceived within the expression or the level of perceived happiness/authenticity of the smile expression. Thus, the use of such scales could be problematic since participants are asked to limit their judgment to two options. Considering that some theoretical models suggest that emotion processing is dimensional rather than categorical, this limitation could be an issue both methodologically and theoretically [30].

A recent study by Roy-Charland et al. [31] compared patterns of judgment made on dichotomous and continuous scales with enjoyment (symmetric Duchenne) and simulated smiles (symmetric non-Duchenne and asymmetric Duchenne). Results of this study revealed that while symmetric Duchenne smiles were always judged as being the happiest/most authentic/most sincere, followed by the asymmetric Duchenne smile, and then the symmetric non-Duchenne smile, subtilities were observed on the continuous scales. In this sense, the average smile judgment ranged between 2 and 5 on a 7-point Likert scale, thus highlighting that participants did not view the smiles as reflecting extreme levels of happiness/authenticity/sincerity or low levels of these characteristics; subtilities which cannot be captured with dichotomous scales. For instance, enjoyment smiles (symmetric Duchenne) were not viewed as extremely happy/sincere/authentic (with scores ranging from 4 to 4.6) and simulated smiles (symmetric non-Duchenne and asymmetric Duchenne) were not viewed as being extremely unhappy/non-sincere/non-authentic (with scores ranging from 1.6 to 4). Thus, these results reiterate the idea of emotion processing that could be dimensional.

Furthermore, some studies suggest that perception and judgment of complex expressions, such as masking smile expressions, may rely more on continuous neural representations of changes in expression along various critical bipolar dimensions such as, but not limited to, negative–positive valence, low–high arousal, and weak–strong dominance [30,32,33,34]. Thus, when it relates to masking smile judgment, it may be a more relevant measure to have participants respond on continuous scales, such as 7-point scales representing low–high levels of happiness/authenticity. Such scales would prevent forced choice responding while also better reflecting the continuity (i.e., lack of discrete emotion boundary categories) of the masking smiles.

In terms of instruction type, it is documented in studies evaluating the impact of instruction on cognitive operations and neural activity that different instructions, including the wording employed (e.g., “happy” or “authentic”) might produce differing results since participants might rely on different processing strategies [35,36,37]. On the other hand, when examining enjoyment and simulated smiles only, it is suggested that different wording (i.e., “happy”, “authentic”, “sincere”) does not produce considerable variations in the expected judgment of smiles [31]. However, regarding masking smiles specifically, this has yet to be explored. Therefore, it would be interesting to understand whether individuals’ judgments regarding the masking smile expressions are processed similarly when the instructions slightly differ. In other words, are there differences in rating when asking participants to make judgments of “happiness” versus judgments of “authenticity”. This is important because in studies of masking smile judgment, the two instruction types (i.e., “really happy” vs. “really authentic”) are often implied to be the same. To the best of our knowledge, no study has examined the impact of varied instruction type in the judgment of masking smiles.

Considering the lack of clarity regarding the use of different methodologies on masking smile judgment and the varying results that have been obtained, the current paper extends the previous work used in masking smile judgment by testing variations in measurement and instruction that have been disregarded in past literature regarding masking smiles. Precisely, the goal of the present study was to explore the judgment of enjoyment and masking smiles using both unipolar and bipolar continuous rating measures and examine differences in the judgment of the same smiles when task instruction varied between making judgments of happiness and authenticity. To do so, two experiments were conducted. The aim of Experiment 1 was to observe the impact of instruction type in the judgment of enjoyment and masking smiles containing traces of anger, fear, sadness, or disgust while using unipolar 7-point rating scales. In Experiment 2, the goal was to examine judgments regarding enjoyment and masking smiles containing traces of anger, fear, sadness, or disgust, when ratings are made on 7-point scales that have bipolar affect labels.

## 2. Experiment 1

The current study explored differences in the judgment of enjoyment smiles and masking smiles containing traces of fear, anger, disgust, and sadness when instructed to rate the expressions on the level of happiness and again on the level of authenticity. Currently, many studies of masking smile judgment have assumed that these two instruction types are synonymous, but little is known about whether these instruction types are perceived synonymously (e.g., [21,22,28,29]). Differences in ratings as a function of instruction type would indicate that enjoyment and masking smiles are not processed identically when instructed to make judgments of happiness versus authenticity, as many current studies of masking smile judgment have assumed. In this experiment, smile ratings were completed on unipolar 7-point scales representing increasing levels of happiness/authenticity to reflect the continuity of the expression and reduce forced choice responding [30,31,33,34,38]. Instruction type was also manipulated within these scales, where participants either had to rate the level of happiness or the level of authenticity of each image.

Based on previous studies [19,20,22], if the scale rating produced similar results to dichotomous responses, it was expected that the enjoyment smiles would be rated more positively (happy and authentic) than the masking smiles while variations in ratings would be observed between the masking smiles. In effect, it could be expected that ratings for smiles with traces of anger in the eyebrows would be rated more positively than other masking smiles and that those with traces of fear would be rated least positively. However, if the response type has an effect on results, other patterns could emerge. With respect to differences in responding as a function of instruction type, no prior studies have specifically compared these two instruction modes regarding the judgment of enjoyment and masking smiles. However, if both instruction types are indeed perceived and judged synonymously, we would not expect a difference in ratings, similar to that found in Roy-Charland et al. [31], in the case of the judgment of enjoyment and simulated smiles.

### 2.1. Method

**Participants.** Thirty-two Caucasian individuals of Canadian nationality (23 females, 9 males, 4 non reported, M_age_ = 20.81, SD = 2.73) with reported normal or corrected-to-normal vision participated in this study. Informed consent was rendered prior to their participation. Power analyses were computed with G Power 3.1.9.7. With a medium effect size (e.g., 0.25), a sample of 24 is sufficient to obtain a power of 0.80.

**Figure 1:**
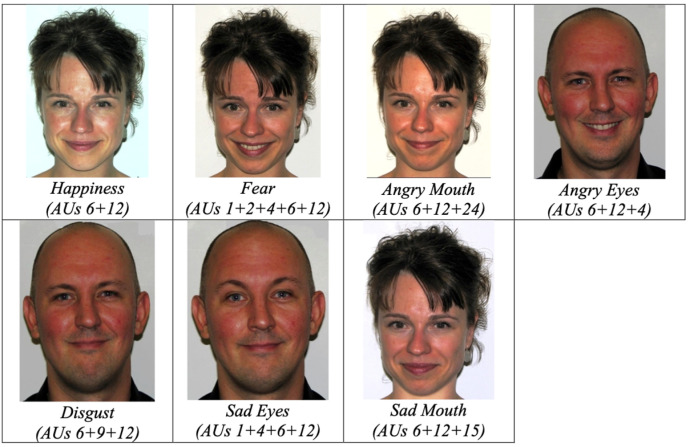
Sample of Stimuli. An example of the happiness smile is presented within the top panel while examples of the six masking smile prototypes are presented within the lower panels from two of the four encoders. The masking smiles entailed characteristics of the happiness smile with additional traces of fear, anger, sadness, or disgust (the muscular activation associated to each emotional expression is presented below the image) produced at an intensity level of B to mirror the subtlety of the activations within micro-expressions.

**Stimuli.** The seven smile prototypes (see Figure 1) were taken from Perron et al. [20] and included one enjoyment smile, which entailed the activation of the Lip Corner Puller (AU12) and the Cheek Raiser (AU6) as described by the Facial Action Coding System (FACS, Ekman et al. [39]), and six masking smiles that contained traces of disgust, fear, anger in the eyes, anger in the mouth, sadness in the eyes, and sadness in the mouth. Each smile type was created in accordance with FACS. To ensure that the subtle parameters of the smile expressions are controlled for (see Gosselin et al. [22] for further explanation), encoders produced the target muscular activations under the guidance of trained and certified FACS coders. The enjoyment smile entailed the activation of AU6 (the Cheek Raiser) and AU12 (the Lip Corner Puller) at intensity level C. The other six smiles contained activations of the enjoyment smile (i.e., AU6 and AU12) as well as traces of anger, sadness, disgust, or fear produced at an intensity level of B to represent the subtleness of micro-expressions (Ekman et al. [39]). Specifically, the fear masking smile contained the additional activations of AU4 (the Brow Lower), AU1 (the Inner Brow Raiser), and AU2 (the Outer Brow Raiser). The angry-eyes masking smile included the additional activation of the AU4 (the Brow Lower), and the angry-mouth masking smiles included the additional activation of AU24 (the Lip Presser). The sad-eyes masking smile contained the activation of both AU4 (the Brow Lower) and AU1 (the Inner Brow Raiser) and the sad-mouth masking smile contained the activation of AU15 (Lip Corner Depressor). Finally, the disgust masking smile contained the additional activation of AU9 (the Nose Wrinkler). The encoders consisted of three Caucasian men and three Caucasian women. The smiles chosen for use in the studies were evaluated by two certified FACS coders and exacted 100% inter-rater agreeability. For each type of smile, four different encoders were used producing a total of 28 pictures.

**Procedure.** The experiment was programmed with the EyeLink Experiment Builder software (https://www.sr-research.com/software/; accessed on 8 June 2024) using a randomized option, greatly reducing the possibility of order effects. Participants were exposed to two experimental blocks, each consisting of 96 images of enjoyment smiles (4 encoders × 12 repetitions) and masking smiles (6 types of smiles × 4 encoders × 2 repetitions). In the first block, participants judged the 96 images on the level of “happiness” on a 7-point scale (0 representing an unhappy smile and 6 representing a happy smile). In the second block, the same participants judged the same 96 images on the level of “authenticity” on a 7-point scale (0 representing an unauthentic smile and 6 representing an authentic smile). Participants were exposed to the stimuli on a computer screen in a counterbalanced order. Once the participants judged the smile, they pressed the space bar on the keyboard and a blank screen allowed the participants time to answer verbally, where the experimenter noted the response. Participants first completed five practice trials to ensure comprehension of the task.

**Data Analyis.** Analyses were completed using the R [4.0.4] package “lme4” [1.1-26] for mixed linear models. Fixed factors were the seven smile prototypes (enjoyment, disgust, angry-eyes, angry-mouth, sad-eyes, sad-mouth, and fear) and two instruction types (happiness vs. authenticity) while participants and encoders were used as random effects.

### 2.2. Results

**Figure 2:**
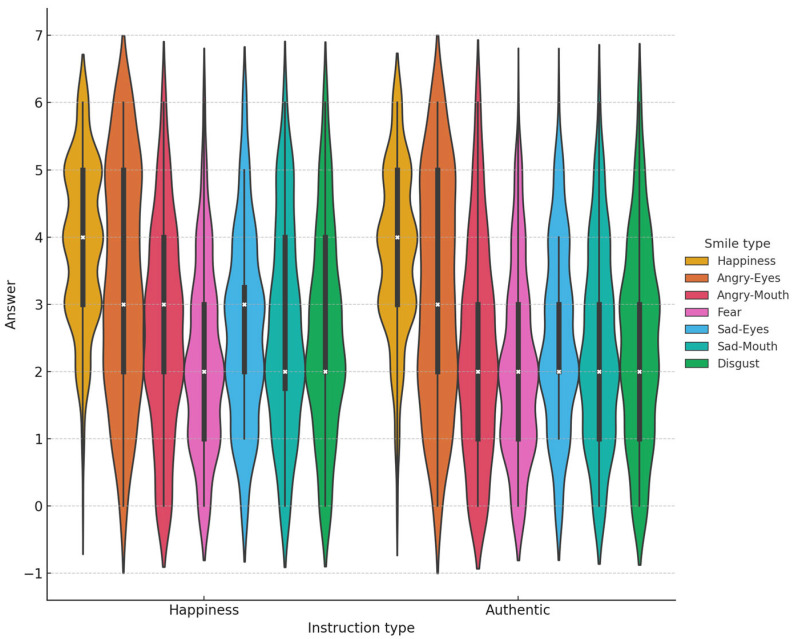
Experiment 1: Distribution of Participant responses by Instruction Type and Smile Type. This figure displays the distribution of participant responses across different instruction types (“Happiness,” “Authentic”) and smile types (“Happiness”, “Angry-Eyes”, “Angry-Mouth”, “Fear”, “Sad-Eyes”, “Sad-Mouth”, and “Disgust”). The x-axis represents participants’ responses on a scale from 0 to 6. The violin plots outline illustrates the kernel probability density (KPD) i.e., the width of each plot reflecting the distribution of responses (wider sections indicate more frequent responses). Inside each violin, box plots depict the median response (white x), interquartile range (middle 50% of the data, within the box), and variability outside the quartiles (the whiskers).

Data are available on the OSF website (see Data Availability Statement). An analysis compared mean ratings on the scales as a function of the seven prototypes (enjoyment, disgust, angry-eyes, angry-mouth, sad-eyes, sad-mouth, and fear) and two instruction types (i.e., happiness vs. authenticity). We ran mixed linear models (LME) with the fixed factors of prototype and instruction type and a random intercept for participants and encoders. Results revealed a main effect of prototype (χ^2^ = 1583.86, *p* < 0.0001) but no effect of instruction (χ^2^ = 1.25, *p* = 0.26), and no interaction (χ^2^ = 2.90, *p* = *0*.82). The enjoyment smile was rated the happiest and most authentic of all the prototypes. The angry-eyes smile was rated significantly happier and more authentic than any other masking smile, but significantly less happy and authentic than the enjoyment smile. The fear smile was rated significantly less happy and authentic than any of the smiles. Figure 2 illustrates the distribution of participant responses by instruction type and smile type. The violin plots were generated using “Seaborn” and “Matplotlib” from Python’s library.

### 2.3. Discussion

Similar to prior studies which used dichotomous response measures [2,19,20,21,40], results indicated that individuals are sensitive to enjoyment and masking smiles as they rated the enjoyment smiles as happier and more authentic than the masking smiles on the 7-point scale. Again, of the masking smiles, those containing traces of fear were rated significantly less happy and authentic and those containing traces of anger in the brows were rated significantly happier and more authentic, consistent with both Perron et al. [20] and Pelot et al. [19]. Interestingly, participants rated the level of happiness and authenticity of the smiles as being similar, indicating that these two instruction modes are processed similarly, like the findings from Roy-Charland et al. [31] for other types of smiles. Further implications will be explored in Section 4 General Discussion.

## 3. Experiment 2

While it has been suggested that dichotomous measures can limit the masking smile judgment by imposing two choices (i.e., “really happy” vs. “not really happy”, “really happy” vs. “pretending to be happy”, etc.) [20,22,38], the results of Experiment 1 do suggest that continuous 7-point measures of low–high levels of happiness/authenticity produce similar results as classic dichotomous measures, reproducing the results of Perron et al. [20]. In addition to asking participants to judge the perceived happiness of the emotion, in the Perron et al. [20] study, participants were also asked to indicate if another emotion was present in the image, where it was shown that participants were less likely to identify accurately the emotion present. Again, in this task, participants were limited to force choice of emotion labels. In a continuum scale where the poles would indicate the presence of a potential emotion (e.g., anger/happiness), it would be interesting to examine if participants would be better at noticing this subtilty, and consequently, influencing their judgment to take account of the trace of a negative emotion.

Thus, the current study examined judgments of enjoyment smiles and masking smile expressions containing traces of fear, anger, disgust, and sadness, but this time when responses were made on 7-point bipolar scales with dimensions between a negative emotion label and happiness (i.e., anger-happiness, fear-happiness, sadness-happiness, and disgust-happiness), in contrast to the unipolar scales used in Experiment 1. As suggested in the literature [30,32,33,34], scales that presents the emotion in bipolar dimensions can better reflect the complexity of the masking smile, considering that it can be characterized as a fusion of negative emotions with a smile. Based on previous studies [19,20,22], it was expected, as in Experiment 1, that the enjoyment smiles would be rated happier than the masking smiles while variations in ratings would be observed between the masking smiles. It could be expected that ratings for smiles with traces of anger in the eyebrows would be rated happier than other masking smiles and that those with traces of fear would be rated least happy. In comparison with Experiment 1, however, we could expect a different pattern in the judgment of the masking smiles, considering the bipolar nature of the scales and the presence of a clue regarding the negative emotion present. In Perron et al. [20], even if smiles with traces of anger in the eyebrows would be rated happier than other masking smiles and that those with traces of fear would be rated least happy, when participants reported the presence of another emotion, they identified anger in the eyebrows most accurately and fear least accurately as the emotion present. Thus, for example, when the bipolar scale comprises anger and happiness, it might give a clue to the participant to the emotion masked, with smiles with traces of anger in the eyebrows reducing the judgment for this particular scale. Nevertheless, it should be noted that no clear hypotheses can be drawn since this is the first exploration of this question with these types of scales.

### 3.1. Method

**Participants.** Thirty-two Caucasian individuals of Canadian nationality (2 males, 30 females, M_age_ = 22.13, SD = 5.15) with reported normal or corrected-to-normal vision participated in this study. Informed consent was obtained prior to participation. Power analyses were computed with G Power 3.1.9.7. With a medium effect size (e.g., 0.25), a sample of 24 is sufficient to obtain a power of 0.80.

**Stimuli.** The seven smile prototypes used in the judgment task (see Figure 1) included one enjoyment smile and six masking smiles containing traces of disgust, fear, anger in the eyes, anger in the mouth, sadness in the eyes, and sadness in the mouth (refer to Experiment 1 for further information on stimuli).

**Procedure.** The experiment was programmed with the EyeLink Experiment Builder software, which includes a randomized default option, greatly reducing the possibility of order effects. Participants rated 96 images of enjoyment smiles (4 encoders × 24 repetitions), and 96 images of masking smiles (6 types of smiles × 4 encoders × 4 repetitions), on perceived level of “happiness” or negative emotion (fear, anger, disgust, or sadness). Participants rated the smiles on 7-point scales ranging from “−3” to “+3”, with “0” being neutral. The negative emotion (fear, sadness, anger, and disgust) was represented on the scale in negative numbers (i.e., −3, −2, and −1) while happiness was represented in positive numbers (i.e., +1, +2, and +3). Half of the participants judged the smiles on scales that began with happiness (i.e., +3 to −3) and the other half judged the smiles on scales that began with the negative emotion dimensions (i.e., −3 to +3) in an effort to observe any possible order bias. Each smile was rated on four scales (anger-happiness, fear-happiness, sadness-happiness, and disgust-happiness). Participants were exposed to the stimuli on a computer screen in a counterbalanced order. The rating scales were presented below the image and participants pressed a button on the keyboard that corresponded to their response on the scale. Prior to beginning the experiment, participants completed five practice trials to ensure comprehension.

**Data Analysis.** Analyses were completed using the R [4.0.4] package “lme4” [1.1-26] for mixed linear models. Fixed factors were the seven smile prototypes (enjoyment, disgust, angry-eyes, angry-mouth, sad-eyes, sad-mouth, and fear) and 4 types of scales (i.e., anger-happiness, fear-happiness, sadness-happiness, and disgust-happiness) and participants and encoders were used as random effects. Simple main effects tests were computed to explore significant interactions and Dunn’s correction was applied to alpha.

### 3.2. Results

**Figure 3:**
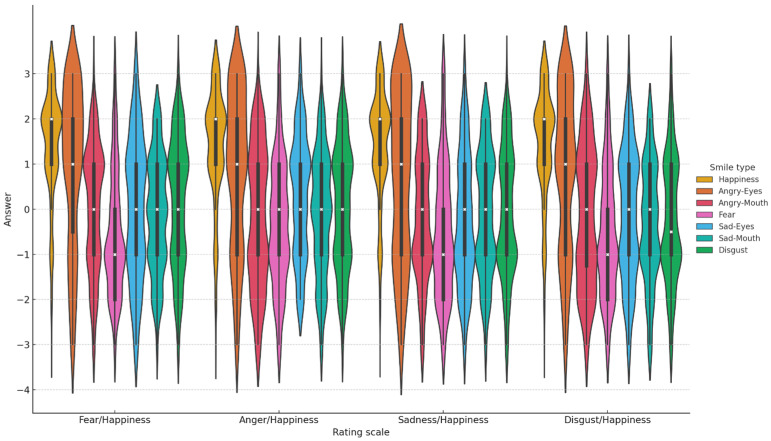
Experiment 2: Distribution of Participant Responses by Scale Type and Smile Type. This figure displays the distribution of participant responses across different scale types (“Fear/Happiness”, “Anger/Happiness”, Sadness/Happiness, Disgust/Happiness) and smile types (“Happiness”, “Angry-Eyes”, “Angry-Mouth”, “Fear”, “Sad-Eyes”, “Sad-Mouth”, and “Disgust”). The x-axis represents participants’ responses on a scale from −3 to +3. The violin plots outline illustrates the kernel probability density (KPD), i.e., the width of each plot reflecting the distribution of responses (wider sections indicate more frequent responses). Inside each violin, box plots depict the median response (white x), interquartile range (middle 50% of the data, within the box), and variability outside the quartiles (the whiskers).

A between-subjects ANOVA revealed no significant effect of order, *F*(1, 30) = 0.11, *p* = 0.74, *η*^2^_p_ = 0.004. There was no difference between smiles judged on the scales that began with the negative emotion dimension versus the scales that began with the happiness dimension. An analysis compared mean ratings on the scales as a function of the seven prototypes (enjoyment, disgust, angry-eyes, angry-mouth, sad-eyes, sad-mouth, and fear) and four scale types (i.e., anger-happiness, fear-happiness, sadness-happiness, and disgust-happiness). We ran mixed linear models (LME) with the fixed factors of prototype and scale type and a random intercept for participants and encoders. Results revealed a main effect of protoype (χ^2^ = 725.45, *p* < 0.0001), but no effect scale type (χ^2^ = 3.88, *p* = 0.27). The interaction was significant (χ^2^ = 29.67, *p* = 0.04). Figure 3 illustrates the distribution of participant responses by scale type and smile type. The violin plots were generated using “Seaborn” and “Matplotlib” from Python’s library.

Simple main effects tests were computed to explore the interaction. Dunn’s correction was applied to alpha and to be considered significant the *p* value needed to be smaller than 0.014. A difference between scales was observed for sad-eyes (*p* < 0.014). Post hoc tests (LSD) revealed that participants judged the sad-eyes more negatively on the sadness-happiness and disgust-happiness scales. A difference between scales was also observed for disgust (*p* < 0.014). Post hoc tests (LSD) revealed that participants judged the disgust smiles more negatively on the disgust-happiness scale than the fear-happiness as well as the anger-happiness scale. For all the other smiles, the differences between scales were not significant, (all *ps >* 0.15). Regarding the ratings between the smile prototypes, post hoc tests (LSD) revealed that the enjoyment smile was rated more positively than all of the prototypes and angry-eyes was rated most positively of the masking smiles. The fear smile was rated significantly more negatively than the enjoyment and masking smiles. However the difference between fear and sad-mouth was only significant for the sadness-happiness scale; where fear was again rated negatively.

### 3.3. Discussion

The results of this experiment revealed that the use of continuous bipolar scales produced similar results as dichotomous response measures and continuous unipolar measures in the judgment of enjoyment and masking smiles containing traces of anger, fear, disgust, and sadness. Specifically, results replicated previous findings and those of Experiment 1, indicating that individuals are sensitive to the enjoyment smiles as they rated them more positively than any of the masking smiles and that those with traces of fear were rated most negatively while those with traces of anger in the eyes were rated more positively [19,20,22].

Thus, it is interesting to note that the use of bipolar scales with dimensions between a negative emotion label and happiness, which presents a clue regarding the emotion that is possibly hidden (e.g., in the anger-happiness scale), did not consistently give participants a clue with regards to the emotion itself. In effect, as an example, smiles with a trace a fear were judged closer to the “sadness” end of the “sadness-happiness” scale than the sad-eyes and sad-mouth smiles. Implications will be further discussed in Section 4 General Discussion.

## 4. General Discussion

Overall, the use of continuous response measures, both with bipolar and unipolar dimensions, did not significantly alter the manner in which enjoyment and masking smile expressions were judged when compared to studies employing dichotomous measures. These findings are similar to Roy-Charland et al. [31] who also found that instruction type and variations in scale type did not produce significant changes in smile judgment. As expected, the enjoyment smiles were rated most positively, happy, and authentic of any smile expression. Further, the patterns of rating of the masking smiles echoed those from both Perron et al. [20] and Pelot et al. [19], whereby masking smiles containing traces of anger presented in the eye were rated most positively, happy, and authentic of the masking smiles, and masking smiles containing traces of fear were rated most negatively, less happy, and less authentic.

**Scale type.** As it relates to the continuous scales with bipolar emotion dimensions, results indicated that the smiles were judged on a positive–negative continuum that was mostly irrespective of the negative emotion label presented on the scale. The lack of differentiation could be due to factors such as insufficient explicit knowledge regarding the expressions’ negative emotion cues, which was proposed in other studies of judgment of enjoyment and masking smile authenticity [20,41]. Nevertheless, although some studies suggest that results of emotional facial expression judgment tasks falter when forced-choice response formats are used [20,22,38], patterns of ratings replicated those from prior studies, validating the use of the continuous response scales in the judgment of enjoyment and masking smiles as well as dichotomous ratings.

**Instruction type.** Relating to varied instruction mode, similar to Roy-Charland et al. [31], ratings did not differ whether participants were instructed to rate the level of happiness or the level of authenticity, indicating that participants perceive and judge enjoyment and masking smiles similarly according to these two instruction types. However, this finding is contradictory to those of other authors [42,43] who suggest that when the emotional content is relevant to the task, instructions may have the potential to influence participants’ responses. In the case of our study, the lack of interaction between the instruction types and the prototypes suggest that judgment between smile types did not differ with these variations of wording. Furthermore, the differences observed in studies examining the judgment of enjoyment and masked smiles cannot be explained by variations in response measures (e.g., dichotomous and continuous measures) or instructions (at least for “happiness” and “authenticity”). Thus, other sources of explanations should be explored in order to explain the different results in the literature. In effect, it would be interesting to examine whether the nature of the stimuli could be a source of explanation of these differences. For instance, other than the methodological differences controlled here, the use of static vs. dynamic stimuli might be another source to explore. Participants might not be sensitive to the traces of masked emotions in dynamic stimuli (e.g., videos) [23,24,25], while those suggesting that individuals are sensitive to those cues employed static images [19,20,22]. Since micro-expressions of the masked emotions can be very brief, participants might not be as sensitive to those sudden movements in the case of a video, in comparison to a static image that displays the cues of the masked expression for a longer period. Additionally, it is also worth exploring whether the presence or intensity of certain facial activations in the stimuli or action units (AUs) [39], could be a source of influence in the results. For example, Perron et al. [20] and Pelot et al. [19] presented AUs at the “B” intensity (intensity varies from A to E in the FACS system) for the traces of negative emotion in their stimuli, while this was not specified in studies suggesting that individuals perform poorly in judging masked emotions [23,24,25]. Thus, it is possible that the intensity of the expression in the latter studies might have been less intense (e.g., “A” intensity). The presence of certain activations could also be worth examining, since some might cause individuals to judge an expression as happier when it is present and less happier when it is absent. While the muscle activation present is detailed in some studies, it is not in all. In order to concretize an explanation for the varying results in literature, these future hypotheses should be considered in future studies.

**Limitations.** A limitation of the studies is that encoders of the expressions were unable to activate the required muscular movements of the face without either opening the mouth or keeping the mouth closed. This limitation is not surprising considering that research indicates the difficulty associated with the voluntary control and activation of facial muscles (e.g., see Gosselin et al. [13]). Nevertheless, future research should ensure equality of the muscular activations of the expressions. Another limitation to the study was that the sample included mostly female participants. However, as it relates to gender differences in the judgment of smile authenticity, literature has failed to observe any reliable effects of gender in adult or child populations (e.g., [28,44]). Thus, future studies should attempt to include equal male and female participants in tasks of judgment and recognition of enjoyment and masking smiles. Furthermore, the arousal of the stimuli was not assessed in this study. Future research could explore this aspect, as it has been suggested that the arousal level of the images might influence emotion judgments (e.g., a negativity bias for high-arousal stimuli) [45].

## 5. Conclusions

In summary, in the present study we showed that the use of bipolar and unipolar dimensional rating scales did not alter the pattern in which enjoyment smiles and masking smiles with traces of anger, fear, sadness, and disgust were perceived when compared to the forced-choice dichotomous measures of previous studies (Experiments 1 and 2). In both studies, participants rated faces with traces of fear significantly less happy and those with traces of anger in the brows were rated significantly happier, consistent with previous literature [19,20]. Additionally, the use of bipolar scales with dimensions between a negative emotion label and happiness (i.e., anger-happiness, fear-happiness, sadness-happiness, and disgust-happiness), were ineffective in influencing the judgment of the masking smile. Participants did not consider the negative emotion possibly present whether the clue referred to the correct masked emotion or not.

## Data Availability

Data for the current study are available open access at: https://osf.io/6sx9b/?view_only=9c5103481a40447fbdaa0bfbf8b32320 (accessed on 6 July 2024).
